# Screening and clinical characteristics analysis of familial hypercholesterolemia in a tertiary public hospital

**DOI:** 10.3389/fcvm.2023.1237261

**Published:** 2023-08-09

**Authors:** Tianzhou Shen, Qingan Fu, Renfei Luo, Yixin Wan, Long Jiang

**Affiliations:** ^1^ Department of Cardiology, The Second Affiliated Hospital of Nanchang University, Nanchang, China; ^2^ Department of Mathematics, Faculty of Natural Sciences, Imperial College London, London, United Kingdom

**Keywords:** familial hypercholesterolemia, screening, prevalence, electronic health records, management strategy

## Abstract

**Background and aims:**

Familial hypercholesterolemia (FH) is becoming a global burden. However, it remains underdiagnosed and undertreated worldwide. This study aimed to observe the screening rate of FH patients and department distribution among hospitalized patients using different diagnostic criteria.

**Methods:**

A total of 45,410 inpatients with LDL-C ≥3.5 mmol/L between 2008 and 2019 were included from The Second Affiliated Hospital of Nanchang University. Inpatients are diagnosed and divided into groups by Dutch Lipid Clinic Network (DLCN) criteria, Chinese-modified DLCN criteria and Chinese expert consensus (CEC) criteria.

**Results:**

There were 172, 1,076 and 115 inpatients included in the DLCN group, Chinese-modified DLCN group and CEC group, respectively (screening rates: 0.38%, 2.37% and 0.25%). These FH patients had a very high risk of atherosclerotic cardiovascular disease (ASCVD) (55.7%–74.4%), especially in the DLCN group and CEC group (70.4%–74.4%). More than half of the patients were in the Department of Cardiology, and other high-risk departments included Neurology, Nephrology, Vascular Surgery, Otolaryngology & Head Neck Surgery and Traditional Chinese Medicine (24.35%–31.51%). Overall, hypertension, coronary heart disease, carotid arteriosclerosis, hepatic cyst, arrhythmia, and nonalcoholic fatty liver disease were common accompanying diseases with FH.

**Conclusions:**

It is necessary to establish appropriate diagnostic criteria and more positive treatment strategies for the FH inpatient population. In addition, promoting awareness of FH among doctors from other departments is also necessary. Therefore, developing a comprehensive management strategy for FH disease is very important.

## Introduction

Familial hypercholesterolemia (FH) is an autosomal dominant disorder characterized by extremely elevated LDL cholesterol (LDL-C) levels, causing severe atherosclerosis and premature coronary heart disease (PCHD) (1). In a meta-analysis involving 7.3 million people, the global prevalence of FH was 1 in 311 (2). China, the world's most populous country, has a significant burden of more than 4.5 million potential FH patients. However, “The Ten Country Study” found that the diagnosis rate of FH in China is extremely low compared to other countries (<0.1%), and doctors in China are less aware of FH guidelines than in Western countries such as the UK (8% vs. 61%), which makes treatment of FH patients extremely difficult. Therefore, scholars urgently need to establish a registration system for patients with FH in China (3). Additionally, “The Ten Country Study” focuses on the awareness of FH in primary care physicians, and the awareness and distribution of FH in other departments besides the Department of Cardiology is not clear at the hospital (4, 5). At present, FH is becoming a global common inherited condition because of its high prevalence and low detection rate (6). The recent FH guidelines emphasize that it is necessary to diagnose and treat FH early to prevent atherosclerotic cardiovascular disease (ASCVD) events (7–9).

The Dutch Lipid Clinic Network (DLCN) criteria are internationally recognized FH diagnostic criteria. Two clinical diagnostic criteria for Chinese FH have been published, including the Chinese-modified DLCN criteria based on the Jiangsu Nutrition Study (10) and the 2018 Chinese expert consensus (CEC) on the screening, diagnosis and treatment of familial hypercholesterolemia (CEC criteria) (11). However, few studies have compared each criterion in the Chinese population (3). In addition, universal screening for FH in China is not yet widespread, and the detection of FH patients mainly relies on cascade screening and is concentrated in the Department of Cardiology. Furthermore, the distribution of FH patients in other departments is unclear, which may cause a large number of FH patients to lose the chance of being diagnosed. Therefore, the purpose of the study is to (1) compare the prevalence rate of FH screened by different diagnostic criteria; (2) evaluate the distribution characteristics of FH patients in different departments in tertiary hospitals in China; (3) provide guidance for the screening of FH in China and other countries and to promote the awareness of FH in departments other than the Department of Cardiology.

## Methods

### Subjects and grouping

The study was a single-center, retrospective study in which clinical information of all inpatients at the Second Affiliated Hospital of Nanchang University from 2008 to 2019 was collected through the hospital's electronic health record (EHR) system. First, we collected adult patients with LDL-C ≥3.5 mmol/L. The patient's LDL-C levels were multiplied by 1.43 if the patient had taken statins before measurement of LDL-C (12).

**Figure 1:**
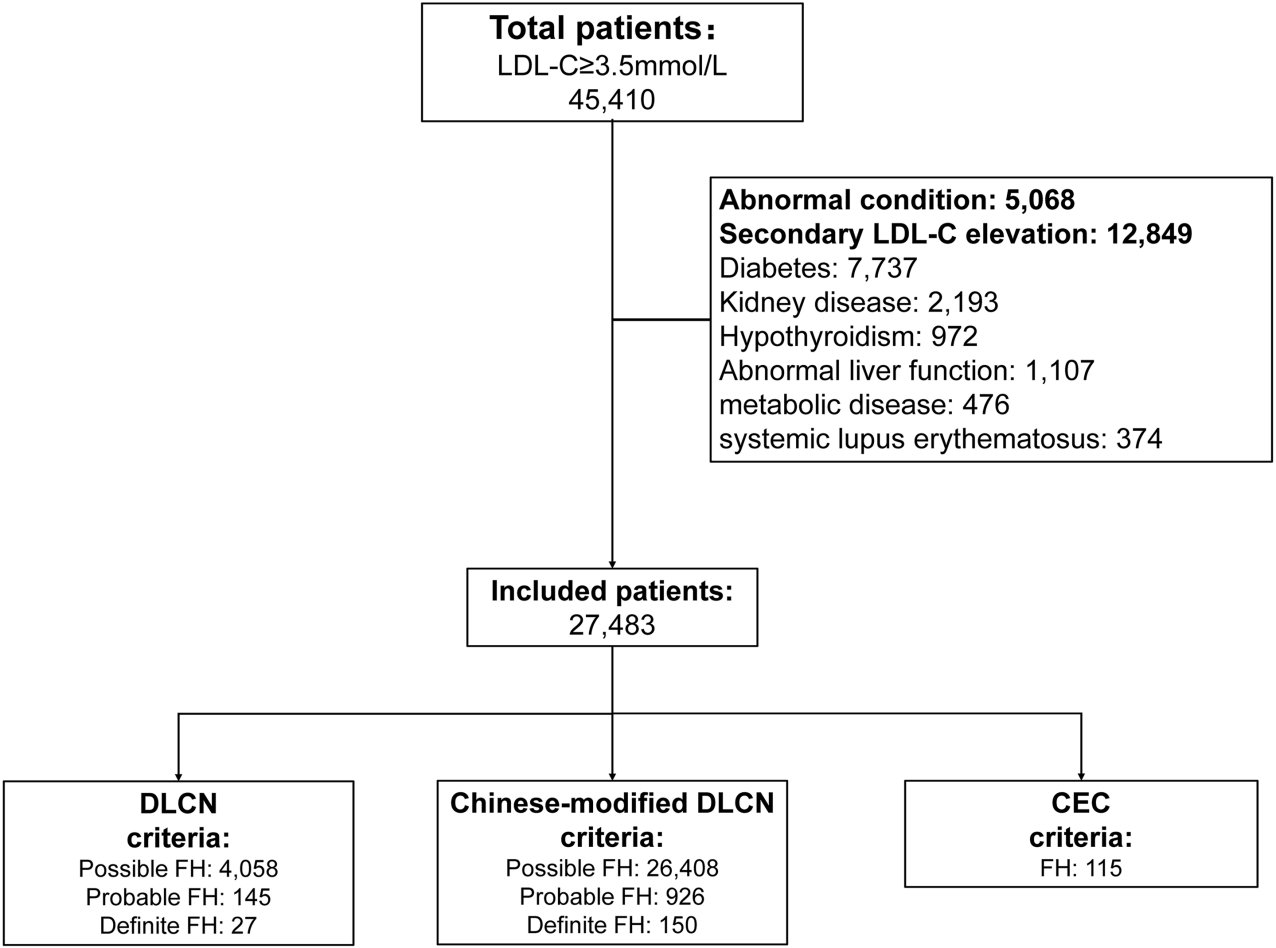
The screening flow chart.

After excluding abnormal conditions and secondary LDL-C evaluation factors, the enrolled patients were divided into the DLCN group (according to DLCN criteria), Chinese-modified DLCN group (according to Chinese-modified DLCN criteria) and Chinese expert consensus (CEC) group (according to CEC criteria) (10–12). Patients meeting the DLCN criteria and Chinese-modified DLCN criteria with probable and definite diagnoses were considered FH patients and were included. The screening flow chart is shown in Figure 1. The study was approved by the Ethics Committee of the Second Affiliated Hospital of Nanchang University (11).

### Exclusion criteria

• Abnormal condition:
  The following conditions were excluded because they may cause abnormal conditions and lead to bias: tumor (cancer, leukemia, treatment with radiation or chemotherapy, etc.), failure (renal failure, liver failure, respiratory failure, multiorgan failure, etc.), shock, and poisoning.
• Secondary LDL-C elevation factors:
  The following conditions were excluded because LDL-C levels are elevated secondarily in these conditions: diabetes, kidney disease (nephrotic syndrome, renal insufficiency, nephritis, membranous nephropathy, acute kidney injury, lga nephropathy, chronic nephrosis, micropathological nephropathy and patients who receive kidney dialysis), hypothyroidism (hypothyroidism, thyroiditis), abnormal liver function (liver insufficiency, abnormal liver function, cirrhosis, liver damage), metabolic disease (hypophysoma, pancreatitis), and systemic lupus erythematosus.

### Diagnostic criteria of FH

• Dutch Lipid Clinic Network criteria (DLCN criteria): This criterion has been published worldwide (Supplementary Table S1) (12).
• Chinese-modified DLCN criteria (Chinese-modified DLCN criteria): family history of first-degree relative with known premature coronary heart disease or vascular disease (1 point); personal history of premature CHD (men <55 years, women <60 years, 2 points) or premature cerebral vascular disease (men <55 years, women <60 years, 1 point); LDL-C higher than 6.0 mmol/L (8 points), 5.0–5.9 mmol/L (5 points), 3.5–4.9 mmol/L (3 points), or 2.5–3.4 mmol/L (1 point). Based on the total score, FH status was classified as definite (>8), probable (6–8), possible (3–5), or unlikely FH (<3) (10).
• Chinese Expert Consensus criteria (CEC criteria): adults meet two of three criteria: (1) LDL-C ≥4.7 mmol/L; (2) tendon xanthomas and/or arcus cornealis (<45 years); and (3) first-degree relative with known FH or premature ASCVD history (men <55 years, women <65 years) (11).

### Clinical data collection

The collected information included demographic characteristics, incidence of disease, blood examination, lipid profile, department distribution and accompanying disease.

Premature ASCVD is defined as premature CHD, cerebrovascular disease, peripheral artery stenosis, and aortic atherosclerotic disease based on each criteria. Statin usage is defined as having used statins before being hospitalized for at least one day.

All of the blood examinations and lipid profiles were selected from the patient's first examination after hospitalization. Blood examination included mean platelet volume, platelet distribution width platelet count, platelet lymphocyte rate (PLR), uric acid, creatinine, glucose, albumin-globulin ratio, low-density lipoprotein cholesterol (LDL-C), apolipoprotein B (ApoB), apolipoprotein A (ApoA), triglyceride (TG), high-density lipoprotein cholesterol (HDL-C), total cholesterol (TC), lipoprotein(a) [Lp(a)].

### Screening rate calculation and statistical analyses

Among inpatients with serum LDL-C above 3.5 mmol/L, the screening rate for each criterion was determined by dividing the number of FH patients in the group by the total number (e.g., the screening rate of the Chinese-modified DLCN criteria: 1,076/45,410 = 2.37%). The screening rate in “high-risk” departments before and after exclusion was determined by dividing the number of FH patients by the number of patients before or after exclusion in the department (e.g., the screening rate in the Department of Cardiology in the Chinese-modified DLCN group: before exclusion: 526/10,843 = 4.85%; after exclusion: 526/8,086 = 6.51%).

Quantitative data with an approximately normal distribution are expressed as *χ* ± s, and ANOVA was used for comparisons between groups. Quantitative data with skewed distribution M (QR) were expressed, the Kruskal–Wallis *H*-test was used for comparisons between multiple groups, and the Mann–Whitney *U*-test was used for two-way comparisons between groups. Statistical data are expressed as frequencies and percentages, and the chi–square test was used for comparisons between groups. Differences were considered statistically significant at *p *< 0.05. All statistical analyses and plotting were conducted in RStudio with R (4.2). Diagrams were plotted by the R package ggplot2 (3.4.1), BioVenn (V1.13), plotrix (V3.8–2), and chorddiag (V0.1.3).

## Result

### Screening rates and clinical characteristics analysis of different criteria

The Second Affiliated Hospital of Nanchang University has a total of 637,807 inpatients from 2008 to 2019. A total of 45,410 adult inpatients (7.12%) with LDL-C ≥3.5 mmol/L were included in the analysis. After excluding patients with abnormal conditions and secondary LDL-C elevation, 27,483 patients were finally included in the analysis. The results show that a total of 145 patients are diagnosed with probable FH and 27 patients with definite FH according to the DLCN criteria (prevalence is 0.38%); a total of 926 patients are diagnosed with probable FH and 150 patients with definite FH according to Chinese-modified DLCN criteria (2.37%); and a total of 115 patients are diagnosed with probable or definite FH according to the 2018 Chinese expert consensus criteria (0.25%). The Venn diagram of each group showed that only 42 patients were included among the three groups (Supplementary Figure S1). In addition, three age groups (18–35, 36–60, >60) were analyzed. Overall, the largest number of patients was in the 36–60 age group (48.8%–69.1%), and the smallest number was in the 18–35 age group (5.2%–9.2%). Furthermore, the number of males was slightly higher in both the 18–35 and 36–60 age groups, and the number of women was slightly higher in the >60 age groups (male: 7.0%–18.3% vs. female: 13.4%–26.7%) (Supplementary Figures S2–S4).

When comparing the clinical characteristics in the three groups, the results showed that there were significant differences (*p *< 0.05) in age, incidence of CHD and premature CHD, mean platelet volume, albumin-globulin ratio, LDL-C, ApoB and TC. Details are shown in Table 1. Overall, the DLCN group had the highest serum lipid level (medium LDL-C: 8.22 mmol/L; TC: 8.41 mmol/L, Lp(a): 36.06 mg/dl) and incidence of CHD (65.7%), and the CEC group had the lowest serum lipid level (medium LDL-C: 5.45 mmol/L; TC: 7.00 mol/L, Lp(a): 25.89 mg/dl). However, the incidence of CHD in the CEC group was slightly higher than that in the Chinese-modified DLCN group (CHD: 39.1% vs. 28.3%, *p *< 0.05 and premature CHD: 33.9% vs. 16.5%, *p *< 0.001). Nearly 50%–60% of patients used statin treatment (DLCN: 59%, Chinese-modified DLCN: 64%, CNC: 43%).

**Table 1:** Clinical characteristics of probable and definite FH in three groups.

		DLCN group *N* = 172	Chinese-modified DLCN group *N* = 1,076	CEC group *N* = 115	*p*
Demographic characteristic	Age years	51 (44,59)***	59 (50,67)	54 (47,64)*	**<0**.**001**
	Gender (male), *n* (%)	98 (57.0)	535 (49.7)	57 (49.6)	0.229
	Familial history	42 (24.4)***	84 (7.8)	115 (100.0)	**nan**
	Statin usage	102 (59.3)	703 (64.1)	50 (43.1)***	**<0**.**001**
Incidence of disease	Hypertension, *n* (%)	46 (26.7)	339 (31.5)	38 (33.0)	0.429
	CHD, *n* (%)	113 (65.7)***	304 (28.3)	45 (39.1)*	**<0**.**001**
	Premature CHD, *n* (%)^a^	98 (57.0)***	178 (16.5)	39 (33.9)***	**<0**.**001**
Blood examination	Mean platelet volume fl	11.1 (10.0,12.2)*	10.7 (9.8,11.8)	10.7 (9.8,11.7)	**0**.**048**
	Platelet distribution width fl	14.3 (12.2,16.3)*	13.7 (11.8,16.0)	14.2 (12.0,16.2)	0.096
	PLR	131.313 (96.045,179.775)	125.698 (95.941,168.125)	123.864 (94.500,165.789)	0.602
	Uric acid umol/L	349.66 (285.90,428.10)	342.21 (281.60,423.70)	342.20 (293.00,409.87)	0.716
	Creatinine umol/L	72.70 (59.99.40,85.54)	71.70 (59.99,83.87)	69.17 (58.72,81.73)	0.389
	Glucose mmol/L	5.07 (4.43,5.75)	5.18 (4.67,5.82)	5.15 (4.60,5.70)	0.261
	Albumin-globulin ratio	1.42 (1.25,1.61)	1.45 (1.29,1.60)	1.49 (1.33,1.71)*	**0**.**018**
Lipid profile	LDL-C mmol/L	8.22 (6.82,9.18)***	6.52 (6.18,7.19)	5.45 (5.01,6.06)***	**<0**.**001**
	ApoB g/L	1.67 (1.41,2.01)***	1.38 (1.20,1.66)	1.31 (1.16,1.49)*	**<0**.**001**
	ApoA g/L	1.07 (0.91,1.25)*	1.10 (0.95,1.29)	1.09 (0.94,1.29)	0.102
	TG mmol/L	1.63 (1.29,2.28)	1.61 (1.24,2.12)	1.60 (1.30,2.05)	0.479
	HDL-C mmol/L	1.15 (0.97,1.33)	1.18 (1.00,1.37)	1.11 (0.95,1.35)	0.148
	TC mmol/L	8.41 (7.24,10.35) ***	7.11 (6.34,8.41)	7.00 (6.19,7.86)**	**<0**.**001**
	Lp(a) mg/dl	36.06 (16.85,64.29)*	28.57 (15.22,50.24)	25.89 (16.34,52.98)	0.074

CHD, coronary heart disease; PLR, platelet lymphocyte rate; ApoB, apolipoprotein B; ApoA, apolipoprotein A; TG, triglyceride; HDL-C, high-density lipoprotein cholesterol; TC, total cholesterol; Lp(a), lipoprotein(a); LDL-C, low-density lipoprotein cholesterol.

Non-normally distributed information is expressed as median (P25, P75).

Bold value indicates *p* < 0.05.

^a^ The premature is defined as man <55 years and women <60 years in DLCN and Chinese-modified DLCN group, and man <55 years and women <65 years in CEC group.

* , **, *** represent *p *< 0.05, *p *< 0.01, *p *< 0.001 respectively in comparison between groups with the Chinese-modified DLCN group.

The incidence of detailed ASCVD is shown in Table 2. Overall, inpatients have a very high prevalence of ASCVD (55.7%–74.4%). The DLCN group and CEC group had a higher occurrence of ASCVD (70.4%–74.4%). The DLCN group had a higher proportion of CHD, while the CEC group had a higher proportion of cerebrovascular disease (CHD: 65.7% vs. 39.1%; ischemic stroke: 4.7% vs. 7.0%; transient ischemic attack: 1.7% vs. 13.0%). The incidence of peripheral artery stenosis and aortic atherosclerotic disease show no significant difference.

**Table 2:** The incidence of ASCVD in FH patients.

	Disease	DLCN group *N* = 172	Chinese-modified DLCN group *N* = 1,096	CEC group *N* = 115	*p*
ASCVD	ASCVD, *n* (%)	128 (74.4)***	599 (55.7)	81 (70.4)**	**<0**.**001**
CHD	CHD, *n* (%)	113 (65.7)***	304 (28.3)	45 (39.1)*	**<0**.**001**
	Premature CHD, *n* (%)^a^	98 (57.0)***	177 (16.4)	39 (33.9)***	**<0**.**001**
	MI, *n* (%)	34 (19.8)***	89 (8.3)	15 (13.0)	**<0**.**001**
	Angina, *n* (%)	35 (20.3)***	94 (8.7)	14 (12.2)	**<0**.**001**
Cerebrovascular disease	IS, *n* (%)	8 (4.7)*	112 (10.4)	8 (7.0)	**0**.**036**
	TIA, *n* (%)	3 (1.7)**	95 (8.8)	15 (13.0)	**0**.**001**
	Carotid artery stenosis, *n* (%)	25 (14.5)	175 (16.3)	26 (22.6)	0.164
Peripheral artery stenosis	Peripheral artery stenosis, *n* (%)	5 (2.9)	19 (1.8)	1 (0.9)	0.423
Aortic atherosclerotic disease	Aortic stenosis, *n* (%)	2 (1.2)	31 (2.9)	3 (2.6)	0.427
	Aortic aneurysm, *n* (%)	0 (0.0)	4 (0.4)	0 (0.0)	nan

MI, myocardial infarction; IS, ischemic stroke; TIA, transient ischemic attack.

Non-normally distributed information is expressed as median (P25, P75).

Aortic stenosis included: aortic stenosis, ulcer dissection and aortic ulcer.

Bold value indicates *p* < 0.05.

^a^ The premature is defined as man <55 years and women <60 years in DLCN and Chinese-modified DLCN group, and man <55 years and women <65 years in CEC group.

* , **, *** represent *p *< 0.05, *p *< 0.01, *p *< 0.001 respectively in comparison between groups with the Chinese-modified DLCN group.

### Distribution and characteristics of FH patients in different departments

**Figure 2:**
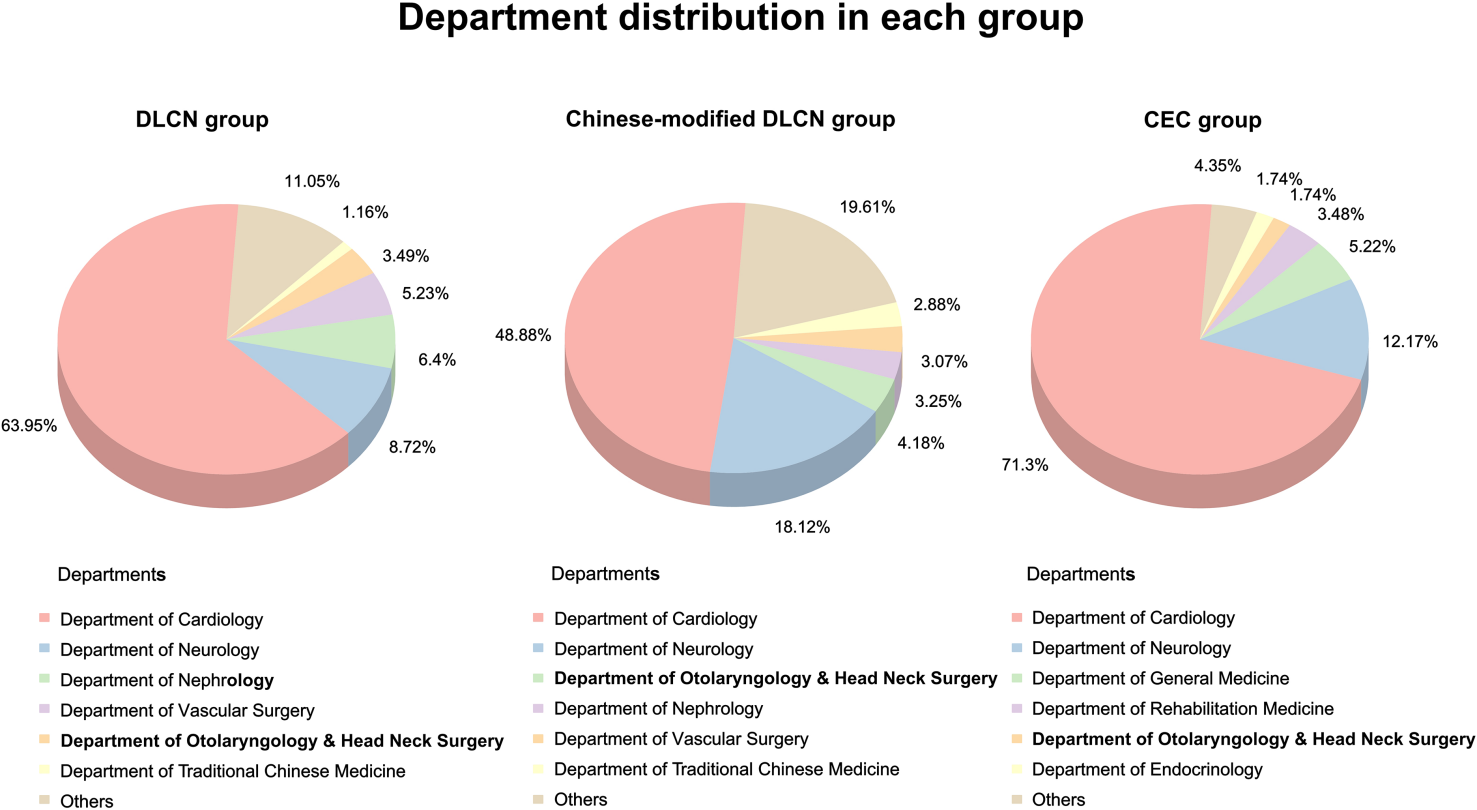
Department distribution of FH patients.

The distribution of FH patients screened by different standards in various departments was analyzed. The results showed that the majority of FH patients were from the Department of Cardiology (48.88%–71.3%, Figure 2), and the second “high-risk” department (8.72%–18.12%) was neurology. Except for the Department of Cardiology, other top departments included the Department of Neurology, Nephrology, Vascular Surgery, Otolaryngology & Head Neck Surgery and Traditional Chinese Medicine, which accounted for more than 25% (24.35%–31.51%) of screened FH patients. The clinical characteristics in each group were divided by “high-risk” departments (Supplementary Tables S2–S4).

The FH screening rates before and after excluding secondary dyslipidemia in each “high-risk” department were analyzed (Table 3). Department of Cardiology, Vascular Surgery and Nephrology had a higher FH screening rate in all three groups. Before exclusion of secondary dyslipidemia, the Department of Cardiology exhibited the highest screening rate (1.01% in the DLCN group, 4.85% in the Chinese-modified DLCN group, and 0.76% in the CEC group). In addition, the screening rate of the Department of Nephrology exhibited great elevation after secondary dyslipidemia exclusion (before: 0.40%; after: 3.35%). The Department of Nephrology even exceeded the Department of Cardiology in DLCN (3.35% vs. 1.36%) and the Chinese-modified DLCN group (10.06% vs. 6.51%).

**Table 3:** The screening rate of FH patients in “high-risk” departments.

Department	Total inpatient		DLCN group			Chinese-modified DLCN group			CEC group		
	Before^a^	After^b^	Number	Before	After	Number	Before	After	Number	Before	After
Cardiology	*N* = 10,843	*N* = 8,086	*N* = 110	1.01%	1.36%	*N* = 526	4.85%	6.51%	*N* = 82	0.76%	1.01%
Neurology	*N* = 6,038	*N* = 4,032	*N* = 15	0.25%	0.35%	*N* = 195	3.23%	4.53%	*N* = 14	0.23%	0.33%
Otolaryngology & head neck surgery	*N* = 3,569	*N* = 3,068	*N* = 6	0.17%	0.20%	*N* = 45	1.26%	1.47%	*N* = 2	0.06%	0.07%
Nephropathy	*N* = 2,743	*N* = 328	*N* = 11	0.40%	3.35%	*N* = 33	1.20%	10.06%	*N* = 1	0.04%	0.30%
Vascular surgery	*N* = 1,347	*N* = 1,133	*N* = 9	0.67%	0.79%	*N* = 35	2.60%	3.09%			
Traditional Chinese medicine	*N* = 1,108	*N* = 733	*N* = 2	0.18%	0.27%	*N* = 31	2.80%	4.23%			

^a^ Means the number of patients before the exclusion of abnormal condition and secondary LDL-C elevation in each department.

^b^ Means the number of patients after the exclusion of abnormal condition and secondary LDL-C elevation in each department.

### Analysis of common accompanying diseases in FH patients

**Figure 3:**
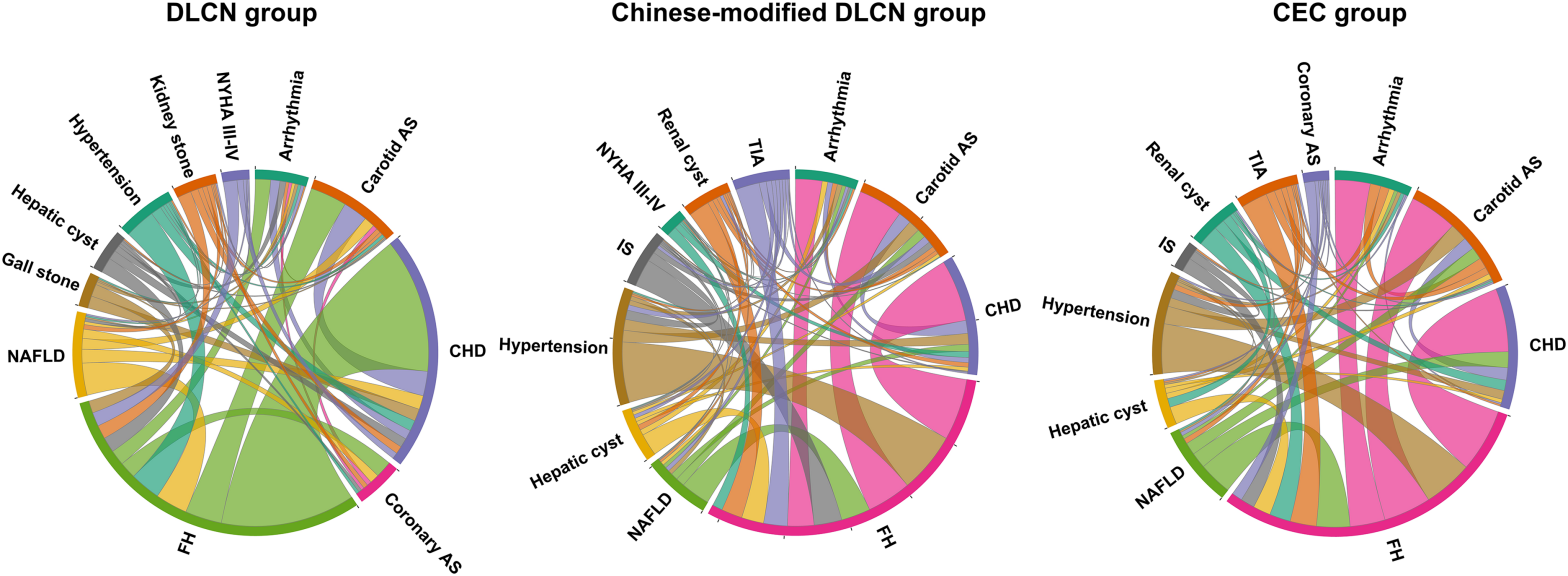
Common accompanying diseases of each group.

The chord diagram of the top 10 accompanying diseases in frequency in each group is shown in Figure 3. Overall, hypertension, CHD, carotid arteriosclerosis (carotid AS), hepatic cyst, arrhythmia, and nonalcoholic fatty liver disease (NAFLD) were the common accompanying diseases with FH in the three groups. In the DLCN group, CHD, carotid AS, NAFLD, hypertension, coronary AS, arrhythmia, hepatic cyst, kidney stone, New York Heart Association Functional Classification III-IV (NYHA III–IV) and gallstone were the top 10 accompanying diseases in frequency. In the Chinese-modified DLCN group, CHD, hypertension, carotid AS, NAFLD, IS, arrhythmia, TIA, hepatic cyst, renal cyst and NYHA III-IV were the top 10 accompanying diseases in frequency. In the Chinese-modified DLCN group, CHD, hypertension, carotid AS, arrhythmia, NAFLD, TIA, renal cyst, hepatic cyst, IS and coronary AS were the top 10 accompanying diseases in frequency. Hypertension was a frequent accompanying disease in each group, but it showed less association with other accompanying diseases.

## Discussion

In this study, we found that the prevalence of FH ranged from 0.42% to 3.99% in hospitalized patients with LDL-C >3.5 mmol/L based on three clinical diagnostic criteria after exclusion of abnormal conditions and secondary LDL-C elevation. The main results are as follows: (1) the prevalence of FH is lower in DLCN criteria and CEC criteria (0.25%–0.38%), and the selected patients are also different. (2) Even with a high prevalence of ASCVD and LDL-C levels in these patients, statin usage is not sufficient. (3) Department of Neurology, Nephrology, Vascular Surgery, Otolaryngology & Head Neck Surgery and Traditional Chinese Medicine are also regarded as “high-risk” departments for FH patients, except for the Department of Cardiology. (4) Hypertension, CHD, carotid AS, hepatic cyst, arrhythmia, and NAFLD are common accompanying diseases of FH. These findings suggest that the diagnosis and treatment of hospitalized FH patients still has a long way to go.

This study found that the screening rates of DLCN criteria, Chinese-modified DLCN criteria and CEC criteria were 0.38%, 2.37% and 0.25%, respectively, in inpatients with LDL-C >3.5 mmol/L, which is the 95th percentile of serum LDL-C in the Chinese population (13). In the China PEACE Million Persons Project, CEC criteria are applied to the largest population, and the prevalence of FH was 0.13% in the 35–70 year age group (14). The reasons for the lower screening rate of the CEC group may be as follows: (1) one of the criteria: LDL-C ≥4.7 mmol/L is much higher than the 95th percentile of LDL-C in China. In addition, the LDL-C levels of FH patients in China are lower than those of patients in Western countries, even if they carry the same LDLR mutation (such as W483X) (15). (2) Family history of ASCVD is difficult to obtain, especially in patients from rural areas who may not be able to distinguish ASCVD by themselves. (3) This study only selected adult inpatients, which may have resulted in the loss of some children FH patients. In addition, another Chinese-modified DLCN criteria also seemed unsuitable for screening, which screened more than 25,000 possible FH patients in this study. Additionally, a large proportion of FH patients admitted to the hospital already have ASCVD (55.7%–74.4%), which suggests that the burden of atherosclerosis in these patients is very severe. Despite the high incidence of ASCVD in these patients, statin usage is relatively low (43.1%–64.1%), emphasizing the need for doctors to intensive lipid-lowering therapy. In addition, only 42 patients can be identified by applying any of the three diagnostic score, implying that the effective detection of an FH patient depends on diagnostic criteria applied by the clinician. Therefore, it is urgent to develop more rigorous clinical diagnosis criteria for FH that meet the needs of the Chinese population and promote early screening, diagnosis and treatment.

One important finding in this study is that except for the Department of Cardiology, the Department of Neurology, Nephrology, Vascular Surgery, Otolaryngology & Head Neck Surgery and Traditional Chinese Medicine are the top departments (accounting for more than 25% of screened FH patients). This result suggested that not only should cardiologists pay attention to FH disease, but doctors from other departments also need to know how to diagnose and treat FH. A study found that the awareness, diagnosis and treatment of FH in cardiologists and primary care physicians were still insufficient, suggesting that more education and training are needed for clinical doctors (16). Systemic atherosclerosis and inflammation in FH patients are more serious than those in normal people, which causes the incidence of cerebral infarction and peripheral vascular atherosclerosis to be higher in these populations (17, 18). Therefore, it is not surprising that some FH patients can be screened in the Department of Neurology and Vascular Surgery. Limited evidence suggests that the Department of Neurology may have a significant role in the diagnosis of hyperlipidemia and in the prevention of vascular disease (19). However, even if patients with a second dyslipidemia disease (such as nephrotic syndrome and renal insufficiency (20, 21) were excluded, the screening rates of FH patients were still higher in the Department of Nephrology. These data may be supported by a certain number of patients with renal arteriosclerosis (22). As a result, it is necessary for nephrologists to focus on finding FH patients. Furthermore, the screening rate of the Department of Otolaryngology & Head Neck Surgery and Traditional Chinese Medicine is relatively low. There are some shunts in FH patients, which may be caused by patients’ selective preference for traditional Chinese medicine and dyslipidemia-induced sudden sensorineural hearing loss (23, 24). These results suggest that attention should also be paid to FH screening in these “high-risk” departments.

This study also analyzed the usual comorbidities associated with FH. In addition to common ASCVD, it was also found that FH was associated with hypertension, NAFLD, arrhythmia and other diseases. Although hypertension is a high incidence FH comorbidity disease, it has a weaker correlation with other comorbidities, which may be due to the high prevalence of hypertension patients in China (25). However, dyslipidemia together with hypertension could aggravate the burden of atherosclerosis (26). In addition, the relationship between FH and NAFLD is unknown. Prior research has indicated that NAFLD has the potential to affect the metabolism of lipoprotein particles and their substituents (27). This can result in dyslipidemia, characterized by elevated TGs, small and dense LDL particles, and reduced levels of HDL-C, thereby increasing the risk of ASCVD (28). Furthermore, a clinical study revealed that patients with FH exhibited a twofold increase in the risk of hospitalization due to heart failure (HF) or atrial fibrillation (AF). In addition, Lp(a) is an independent causal risk factor for ASCVD (29). This study also found that serum Lp(a) level is elevated in FH patients (medium: 36.06–25.89 mg/dl). Recent studies had found there is −2 fold increased ASCVD risk in FH combined with elevated Lp(a) levels when compared to FH alone (30). Therefore, other diseases and risk factors in FH patients also need doctor's attention.

Research on FH started relatively late in China compared with other countries. However, China has made great efforts to conduct studies on the diagnosis and treatment of FH in recent years. In 2018, HoFH was selected into the First Batch of Rare Diseases Catalog, and a Chinese expert consensus on the screening and treatment of familial hypercholesterolemia was published (11, 31). In 2021, the “14th Five-Year Plan” National Key Research and Development Program of China launched a program that focuses on screening and intervention for hypercholesterolemia in China, one of which is to establish diagnostic criteria for FH in the Chinese population (32). On January 1, 2022, a PCSK9 (proprotein convertase subtilisin type 9) inhibitor was added to the national medical insurance directory, and the price was significantly reduced (−40$ one dose), which will bring huge benefits to FH patients. In conclusion, with an increasing number of projects and studies about FH carried out, the screening and diagnosis rates of FH will be significantly improved in the near future.

### Limitations

The study has the following limitations: (1) for study convenience, patients with LDL-C >3.5 mmol/L were selected, which is the 95th percentile of serum LDL-C levels in adults (13). This may miss some patients who have taken statins or received other lipid-lowering treatments. (2) All of these patients are missing genetic diagnosis information, which may not be accurate (33). (3) The study is a retrospective study, so the tendon xanthomas and arcus cornealis feature in first-degree relatives of patients could not be obtained. Therefore, this may reduce the number of patients included in DLCN group and CEC group. (4) Patients with secondary hyperlipidemia, such as diabetes, were excluded, and the LDL-C value of these patients before diabetes and other diseases is lacking, which may cause missing some FH patients. (5) The study is a single-center study that has its own shortcomings, which may limit the effectiveness of data extrapolation in other countries and populations. (6) The low FH screening rates in some departments may be due to the fact that lipid testing is not used as a routine admission test which may miss some patients. However, 45,410 patients were included in the screening, and all patients’ first postadmission LDL-C measurements were obtained for the diagnosis of FH in this study, reducing bias introduced by directly using EHR codification. In addition, The abnormal condition and secondary LDL-C elevation condition were strictly excluded according to diagnosis to reduce bias.

## Conclusion

Our study may provide some evidence for the national FH screening of inpatients. The study hints that the diagnostic criteria for screening FH among hospitalized patients are still incomplete. In addition, other departments, such as nephrology, nephrology and vascular surgery, still have a great number of FH patients who may be underdiagnosed and undertreated. Therefore, it is necessary not only to strengthen the understanding of FH disease among doctors in these “high-risk” departments but also to develop complete screening and management strategies for FH inpatients.

## Data Availability

The data used and/or analyzed during the current study is available from the corresponding author on reasonable request.
