# Supplementary material for: Screening and clinical characteristics analysis of familial hypercholesterolemia in a tertiary public hospital
Source: Front Cardiovasc Med. 2023 Aug 9;10:1237261. doi: 10.3389/fcvm.2023.1237261 (PMC10445126; doi:10.3389/fcvm.2023.1237261)
Supplement: Supplementary file 1 [file Datasheet1.docx]

**Screening and clinical characteristics analysis of familial hypercholesterolemia in a tertiary public hospital**

Tianzhou Shen^1†^, Qingan Fu^1†^, Renfei Luo^1^, Yixin Wan ^2^, Long Jiang ^1*^

† The authors equally contributed to this work

* Corresponding authors

**Supplementary Data**

**Table S1 Dutch Lipid Clinic Network diagnostic criteria**

**Table S2 Department classification of the Second Affiliated Hospital of Nanchang**

**Table S3 Clinical characteristics of each department in DLCN groups**

**Table S4 Clinical characteristics of each department in Chinese-modified DLCN groups**

**Table S5 Clinical characteristics of each department in Chinese-modified DLCN groups**

**Figure S1 overlap of different groups**

**Figure S2 age-sex distribution in DLCN group**

**Figure S3 age-sex distribution in Chinese-modified DLCN group**

**Figure S4 age-sex distribution in CEC group**

**Table S1 Dutch Lipid Clinic Network diagnostic criteria**

| **Category** | **Points** |
| --- | --- |
| **Family history** |  |
| First-degree relatives with known premature* coronary heart disease (CHD) | 1 |
| First-degree relatives with known elevated LDL-C levels | 1 |
| First-degree relatives with tendon xanthomas and/or arcus cornealis OR | 2 |
| Children aged less than 18 years with known elevated LDL-C levels | 2 |
| **Personal history** |  |
| Premature CAD | 2 |
| Premature cerebral or peripheral vascular disease | 1 |
| **Physical examination** |  |
| Tendon xanthomas | 6 |
| Arcus cornealis prior to age 45 years | 4 |
| **LDL-C level (mmol/L)** |  |
| ＞8.5 | 8 |
| 6.5-8.4 | 5 |
| 5.0-6.4 | 3 |
| 4.0-4.9 | 1 |
| **Genetic analysis** |  |
| Functional mutation in the LDLR, apo B or PCSK9 gene | 8 |
| **Diagnosis (diagnosis is based on the total number of points obtained)** |  |
| Definite FH | >8 |
| Probable FH | 6-8 |
| Possible FH | 3-5 |
| Unlikely FH | <3 |

FH, familial hypercholesterolemia; LDL-C, low density lipoprotein cholesterol; LDLR, low density lipoprotein receptor; apoB, apolipoprotein B; PCSK9, proprotein convertase subtilisin/kexin type 9.

*Premature referred to <55 years for men; <60 years for women.

**Table S2 Department classification of the Second Affiliated Hospital of Nanchang**

| Internal medicine | Surgery | Ophthalmology and Otorhinolaryngology | Department of Emergency |
| --- | --- | --- | --- |
| Department of Respiration | Department of Vascular Surgery | Department of Ophthalmology | Department of Emergency |
| Department of Traditional Chinese Medicine | Department of Hepatobiliary Surgery | Department of Orthopaedics |  |
| Department of Oncology | Department of Neurosurgery | Department of Otolaryngology & Head Neck Surgy |  |
| Department of Urology | Department of Thyroid Surgery |  |  |
| Department of General Medicine | Department of Gastrointestinal Surgery |  |  |
| Department of Hepatology | Department of Cardiovascular Surgery |  |  |
| Department of Rheumatology and Immunology | Department of Thoracic Surgery |  |  |
| Department of Haematology | Department of Breast Surgery |  |  |
| Department of Rehabilitation Medicine | Department of Plastic Surgery |  |  |
| Department of Obstetrics and Gynaecology | Department of Transplantation |  |  |
| Department of Stomatology | Department of Orthopedics |  |  |
| Department of Painology |  |  |  |
| Department of Dermatology |  |  |  |
| Department of Infectious Diseases |  |  |  |
| Department of Paediatrics |  |  |  |
| Department of Endocrinology |  |  |  |
| Department of Nephrology |  |  |  |
| Department of Neurology |  |  |  |
| Department of Cardiology |  |  |  |
| Department of Gastroenterology |  |  |  |

**Table S3 Clinical characteristics of each department in DLCN groups**

|  |  | Department of Cardiology  (n=110) | Department of Neurology  (n=15) | Department of Nephrology  (n=11) | Department of Vascualr Surgery  (n=9) | Department of Otolaryngology & Head Neck Surgy (n=6) | Department of Triditional Chinese Medicine  (n=2) | P value |
| --- | --- | --- | --- | --- | --- | --- | --- | --- |
| General characteristic | Age years | 49.964(11.633) | 61.867(13.569) | 45.909(16.301) | 58.333(12.401) | 46.000(8.145) | 54(13.000) | 0.004 |
|  | Gender (male), n (%) | 71(64.6) | 6(40.0) | 7(63.6) | 4(44.4) | 3(50.0) | 1(50.0) | 0.447 |
|  | Familial history | 31(28.2) | 4(26.7) | 1(9.1) | 0(0.0) | 1(16.7) | 0(0.0) | nan |
|  | Statin usage | 66(60.0) | 13(86.7) | 3(27.3) | 4(44.4) | 3(50.0) | 2(100.0) | nan |
| History | Hypertension, n (%) | 32(29.1) | 5(33.3) | 1(9.1) | 3(33.3) | 0(0.0) | 0(0.0) | nan |
|  | CHD, n(%) | 102(92.7) | 0(0.0) | 0(0.0) | 0(0.0) | 4(66.7) | 1(50.0) | nan |
|  | Premature CHD, n(%) ^a^ | 90(81.2) | 0(0.0) | 0(0.0) | 0(0.0) | 4(66.7) | 0(0.0) | nan |
| Blood examination | Mean platelet volume fl | 11.1(1.568) | 11.3(1.492) | 11.6(0.942) | 10.5(1.530) | 12.7(1.159) | 11.6(0.850) | 0.31 |
|  | Platelet distribution width fl | 14.5(12.5,16.4) | 13.7(13.3,14.6) | 15.4(13.1,15.8) | 14.1(12.2,16.3) | 15.8(13.6,18.2) | 11.9(11.9,17.1) | 0.91 |
|  | PLR | 122.3(93.7,159.8) | 181.4(135.8,241.3) | 127.(89.2,177.9) | 103.6(81.6,181.3) | 158.3(155.4,330.7) | 91.4(91.4,113.1) | 0.057 |
|  | Uric acid umol/L | 358.8(306.4,446.3) | 365.5(300.9,396.0) | 339.5(273.2,415.7) | 377.5(285.3,428.1) | 285.2(240.8,308.6) | 325.4(325.4,335.5) | 0.342 |
|  | Creatinine umol/L | 74.3(63.8,85.7) | 67.8(54.7,86.5) | 84.(59.8,89.4) | 77.88(59.3,84.1) | 69.7(55.7,73.8) | 64.4(64.4,91.7) | 0.599 |
|  | Glucose mmol/L | 5.1(4.5,5.8) | 4.5(4.4,5.6) | 4.3(3.8,5.5) | 4.8(4.3,6.1) | 5.3(5.2,5.9) | 4.4(4.4,5.8) | 0.291 |
|  | Albumin-globulin ratio | 1.49(0.3) | 1.39(0.25) | 1.13(0.2) | 1.29(0.33) | 1.54(0.17) | 1.52(0.23) | 0.003 |
| Lipid | LDL-C mmol/L | 7.25(6.62,8.72) | 8.90(8.72,9.60) | 9.64(8.91,11.01) | 8.65(8.51,9.13) | 7.44(6.82,8.98) | 8.65(8.6519.44) | <0.001 |
|  | ApoB g/L | 1.55(1.30,1.85) | 1.65(1.61,1.9) | 2.31(2.14,2.96) | 2.01(1.82,2.14) | 1.52(1.37,2.06) | 1.77(1.77,1.94) | <0.001 |
|  | ApoA g/L | 1.04(0.89,1.18) | 1.00(0.93,1.19) | 1.42(1.10,1.71) | 1.01(0.89,1.13) | 1.23(1.19,1.3) | 1.0(1.00,1.07) | 0.037 |
|  | TG mmol/L | 1.58(1.24,2.15) | 1.18(1.07,1.83) | 2.48(1.83,3.17) | 1.66(1.42,1.94) | 1.32(0.87,1.81) | 0.88(0.88,1.59) | 0.016 |
|  | HDL-C mmol/L | 1.08(0.94,1.29) | 1.19(1.04,1.46) | 1.43(1.04,1.76) | 1.19(0.98,1.28) | 1.32(1.29,1.35) | 1.08(1.08,1.29) | 0.038 |
|  | TC mmol/L | 7.95(6.84,9.03) | 8.91(8.41,10.35) | 13.33(11.20,15.52) | 10.76(8.43,11.62) | 7.78(7.24,10.72) | 8.27(8.27,8.49) | <0.001 |
|  | Lp(a) mg/dL | 31.90(16.20,52.98) | 33.51(12.42,40.70) | 115.64(46.80,151.60) | 71.55(66.42,95.58) | 39.02(21.76,46.70) | 22.16(22.16,26.32) | 0.003 |

CHD: coronary heart disease, PLR: platelet lymphocyte rate, ApoB: apolipoprotein B, ApoA: apolipoprotein A, TG: triglyceride, HDL-C: high-density lipoprotein cholesterol, TC: total cholesterol, Lp(a): lipoprotein(a), LDL-C: low-density lipoprotein cholesterol.

^a^ the premature is defined as man <55 years and women < 60 years.

* Represents P<0.01, P<0.001 respectively in comparison between groups with the Chinese-modified DLCN group.

** Represents P<0.01respectively in comparison between groups with the Chinese-modified DLCN group.

*** Represents P<0.001 respectively in comparison between groups with the Chinese-modified DLCN group.

**Table S4 Clinical characteristics of each department in Chinese-modified DLCN groups**

|  |  | Department of Cardiology  (n=526) | Department of Neurology  (n=195) | Department of Otolaryngology & Head Neck Surgery  (n=45) | Department of  Vascular Surgery  (n=35) | Department of Nephrology  (n=33) | Department of Triditional Chinese Medicine  (n=31) | P value |
| --- | --- | --- | --- | --- | --- | --- | --- | --- |
| General characteristic | Age years | 58 (49,66) | 64(53,72) | 53(44,58) | 64(57,74) | 47(37,60) | 60(55,66) | <0.001 |
|  | Gender (male), n (%) | 278(52.852) | 101(51.795) | 2(44.444) | 2(57.143) | 19(57.576) | 9(29.032) | 0.132 |
|  | Familial history | 64(12.167) | 7(3.59) | 1(2.222) | 0 | 1(3.03) | 1(3.226) | nan |
|  | Statin usage | 369(70.152) | 156(80.00) | 11(24.444) | 17(48.571) | 11(33.333) | 22(70.968) | <0.001 |
| History | Hypertension, n (%) | 167(31.749) | 89(45.641) | 7(15.556) | 12(34.286) | 5(15.152) | 7(22.581) | <0.001 |
|  | CHD, n(%) | 272(51.711) | 4(2.051) | 5(11.111) | 2(5.714) | 0 | 2(6.452) | nan |
|  | Premature CHD, n(%)^a^ | 161(30.61) | 1(0.513) | 5(11.111) | 1(2.857) | 0 | 1(3.226) | nan |
| Blood examination | Mean platelet volume fl | 10.7(9.9,11.0) | 10.4(9.6,11.5) | 11.2(10.2,12.1) | 10.7(9.9,11.7) | 11.6(10.5,12.2) | 10.5(9.7,11.6) | 0.011 |
|  | Platelet distribution width fl | 13.9(12.0,16.2) | 14.2(11.8,16.1) | 13.6(11.7,15.7) | 12.7(11.3,15.8) | 14.4(12.1,15.8) | 12.6(10.9,15.4) | 0.189 |
|  | PLR | 116.2(93.0,157.8) | 135.8(105.1,175.6) | 141.3(99.3,198.4) | 143.2(92.1,181.3) | 123.1(102.7,168.6) | 122.1(113.1,171.3) | 0.003 |
|  | Uric acid umol/L | 358.8(299.9,436.3) | 326.9(259.8,408.4) | 285.2(259.7,351.7) | 342.2(255.1,418.1) | 339.5(297.3,423.7) | 316.4(277.1,365.6) | <0.001 |
|  | Creatinine umol/L | 73.7(62.1,85.7) | 71.3(60.9,82.8) | 66.0(58.6,75.4) | 74.1(59.3,88.9) | 76.1(62.8,88.3) | 64.4(57.2,76.9) | 0.019 |
|  | Glucose mmol/L | 5.2(4.7,5.8) | 5.1(4.5,5.6) | 5.8(4.9,7.4) | 4.9(4.6,5.9) | 4.9(4.0,5.8) | 5.5(5.0,6.0) | <0.001 |
|  | Albumin-globulin ratio | 1.46(1.31,1.62) | 1.49(1.34,1.63) | 1.43(1.25,1.51) | 1.34(1.20,1.63) | 1.25(1.08,1.44) | 1.39(1.36,1.54) | <0.001 |
| Lipid | LDL-C mmol/L | 6.45(6.15,7.02) | 6.45(6.15,7.04) | 6.81(6.39,7.53) | 6.61(6.12,7.87) | 7.52(6.69,8.91) | 6.56(6.51,7.19) | <0.001 |
|  | ApoB g/L | 1.31(1.16,1.57) | 1.27(1.16,1.56) | 1.66(1.37,2.07) | 1.48(1.21,2.01) | 1.77(1.52,2.14) | 1.33(1.21,1.64) | <0.001 |
|  | ApoA g/L | 1.09(0.93,1.27) | 1.08(0.94,1.28) | 1.13(0.98,1.25) | 1.13(0.97,1.26) | 1.44(1.20,1.71) | 1.07(1.00,1.22) | <0.001 |
|  | TG mmol/L | 1.62(1.25,2.10) | 1.44(1.12,1.88) | 1.44(0.87,2.25) | 1.47(1.25,2.00) | 2.04(1.65,3.05) | 1.59(1.09,2.17) | <0.001 |
|  | HDL-C mmol/L | 1.15(0.98,1.34) | 1.16(0.99,1.35) | 1.30(1.14,1.39) | 1.23(0.94,1.41) | 1.42(1.19,1.82) | 1.27(1.16,1.37) | <0.001 |
|  | TC mmol/L | 6.76(6.23,7.86) | 6.66(6.13,7.73) | 8.57(7.69,9.83) | 8.30(6.72,9.84) | 10.44(8.79,11.32) | 7.13(6.49,8.38) | <0.001 |
|  | Lp(a) mg/dL | 27.60(14.80,47.34) | 24.36(15.70,40.99) | 36.98(21.23,61.03) | 59.19(20.71,95.04) | 69.06(21.57,118.96) | 26.32(17.98,41.37) | <0.001 |

CHD: coronary heart disease, PLR: platelet lymphocyte rate, ApoB: apolipoprotein B, ApoA: apolipoprotein A, TG: triglyceride, HDL-C: high-density lipoprotein cholesterol, TC: total cholesterol, Lp(a): lipoprotein(a), LDL-C: low-density lipoprotein cholesterol.

^a^ the premature is defined as man <55 years and women < 60 years.

* Represents P<0.01, P<0.001 respectively in comparison between groups with the Chinese-modified DLCN group.

** Represents P<0.01respectively in comparison between groups with the Chinese-modified DLCN group.

*** Represents P<0.001 respectively in comparison between groups with the Chinese-modified DLCN group.

**Table S5 Clinical characteristics of each department in CEC groups**

|  |  | Department of Cardiology  (n=82) | Department of Neurology  (n=14) | Department of General Medicine  (n=6) | Department of Rehabilitation Medicine  (n=4) | Department of Otolaryngology & Head Neck Surgery  (n=2) | Department of Endocrinology  (n=2) | P value |
| --- | --- | --- | --- | --- | --- | --- | --- | --- |
| General characteristic | Age years | 54(11.812) | 62(13.601) | 53(8.807) | 56(5.874) | 49(11.00) | 32(1.00) | 0.031 |
|  | Gender (male), n (%) | 42(51.22) | 8(57.143) | 2(33.333) | 0 | 0 | 1(50.00) | nan |
|  | Familial history | 82(100.0) | 14(100.0) | 6(100.0) | 4(100.0) | 2(100.0) | 2(100.0) | 1 |
|  | Statin usage | 36(43.9) | 7(50.0) | 3(50.0) | 1(25.0) | 2(100.0) | 0 | nan |
| History | Hypertension, n (%) | 27(32.9) | 7(50.0) | 1(16.7) | 1(25.0) | 0 | 0 | nan |
|  | CHD, n(%) | 41(50.0) | 0 | 1(16.7) | 1(25.0) | 0 | 1(50.0) | nan |
|  | Premature CHD, n(%)^a^ | 35(42.7) | 0 | 1(16.7) | 1(25.0) | 0 | 1(50.0) | nan |
| Blood examination | Mean platelet volume fl | 10.8(1.429) | 9.9(0.868) | 11.8(1.507) | 10.1(1.629) | 9.1(0.10) | 10.7(0.75) | 0.045 |
|  | Platelet distribution width fl | 14.1(12.2,16.2) | 15.6(13.5,16.2) | 13.1(12.6,17.3) | 11.6(9.6,11.6) | 9.7(9.7,16.0) | 10.6(10.6,13.6) | 0.338 |
|  | PLR | 125.8(97.7,153.6) | 123.9(93.0,210.9) | 81.0(810.,97.9) | 128.2(119.0,128.1) | 173.4(173.4,708.8) | 85.0(85.0,155.4) | 0.086 |
|  | Uric acid umol/L | 352.0(298.1,415.4) | 329.9(282.0,388.7) | 304.7(283.4,437.2) | 335.8(281.9,335.8) | 259.3(259.3,322.7) | 240.8(240.8,297.9) | 0.31 |
|  | Creatinine umol/L | 70.3(60.4,84.7) | 67.4(58.7,72.0) | 63.3(56.5,72.6) | 58.3(58.3,58.3) | 54.7(54.7,73.3) | 48.6(48.6,73.8) | 0.453 |
|  | Glucose mmol/L | 5.2(4.6,5.8) | 5.0(4.6,5.3) | 4.9(4.8,5.0) | 5.7(5.7,5.7) | 4.6(4.6,4.9) | 5.5(5.5,5.7) | 0.295 |
|  | Albumin-globulin ratio | 1.48(1.30,1.73) | 1.51(1.46,1.71) | 1.50(1.35,1.56) | 1.56(1.37,1.56) | 1.52(1.52,1.83) | 1.34(1.34,1.52) | 0.877 |
| Lipid | LDL-C mmol/L | 5.29(5.01,5.96) | 5.85(5.52,6.56) | 5.58(4.83,6.96) | 5.28(5.12,5.28) | 6.98(6.98,9.61) | 4.80(4.80,8.98) | 0.065 |
|  | ApoB g/L | 1.27(1.13,1.48) | 1.26(1.22,1.46) | 1.26(1.10,1.50) | 1.76(1.44,1.76) | 1.407(1.407,1.48) | 1.13(1.13,2.06) | 0.784 |
|  | ApoA g/L | 1.11(0.31) | 1.08(0.177) | 1.12(0.157) | 1.25(0.169) | 1.14(0.196) | 1.21(0.02) | 0.933 |
|  | TG mmol/L | 1.55(1.24,1.99) | 1.37(1.07,1.83) | 1.85(1.74,2.45) | 2.55(2.51,2.55) | 1.35(1.35,2.77) | 1.14(1.14,1.32) | 0.049 |
|  | HDL-C mmol/L | 1.09(0.94,1.36) | 1.11(1.04,1.20) | 1.01(0.94,1.21) | 1.22(1.11,1.22) | 0.93(0.93,1.45) | 1.32(1.32,1.50) | 0.73 |
|  | TC mmol/L | 6.88(1.215) | 7.14(0.858) | 7.41(1.594) | 7.10(1.00) | 7.87(0.265) | 8.57(2.155) | 0.337 |
|  | Lp(a) mg/dL | 24.38(16.20,45.62) | 35.69(23.02,53.85) | 13.95(10.65,40.60) | 92.50(90.93,92.50) | 22.20(22.20,92.52) | 20.84(20.84,46.70) | 0.096 |

CHD: coronary heart disease, PLR: platelet lymphocyte rate, ApoB: apolipoprotein B, ApoA: apolipoprotein A, TG: triglyceride, HDL-C: high-density lipoprotein cholesterol, TC: total cholesterol, Lp(a): lipoprotein(a), LDL-C: low-density lipoprotein cholesterol.

^a^ the premature is defined as man <55 years and women < 65 years.

* Represents P<0.01, P<0.001 respectively in comparison between groups with the Chinese-modified DLCN group.

** Represents P<0.01respectively in comparison between groups with the Chinese-modified DLCN group.

*** Represents P<0.001 respectively in comparison between groups with the Chinese-modified DLCN group.


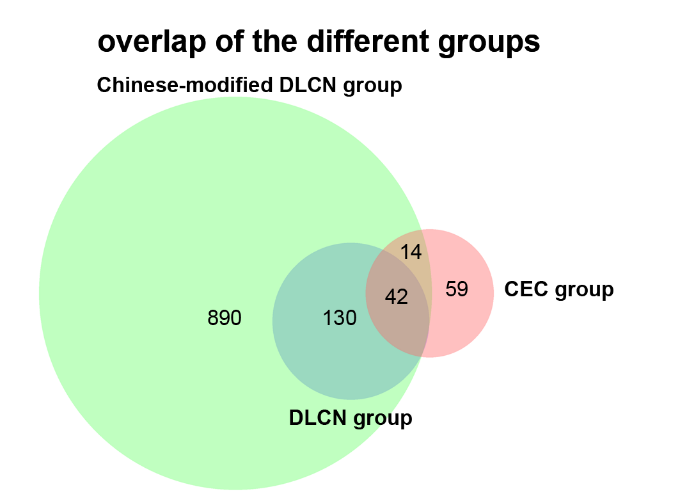


**Figure S1 overlap of different groups**


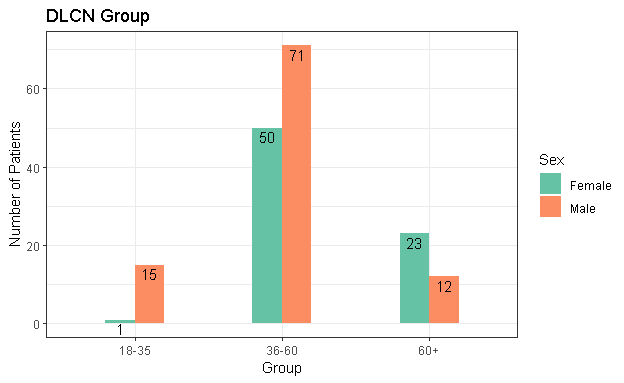


**Figure S2 age-sex distribution in DLCN group**


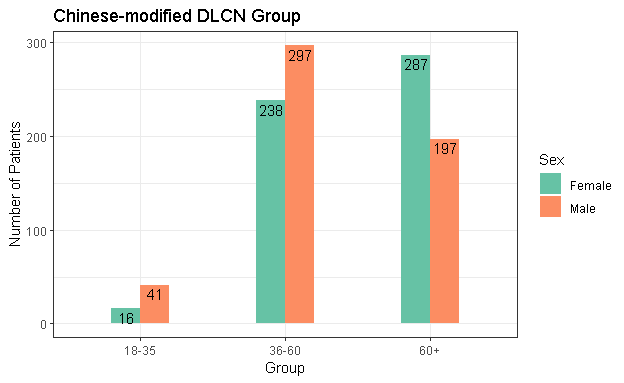


Figure S3 age-sex distribution in Chinese-modified DLCN group


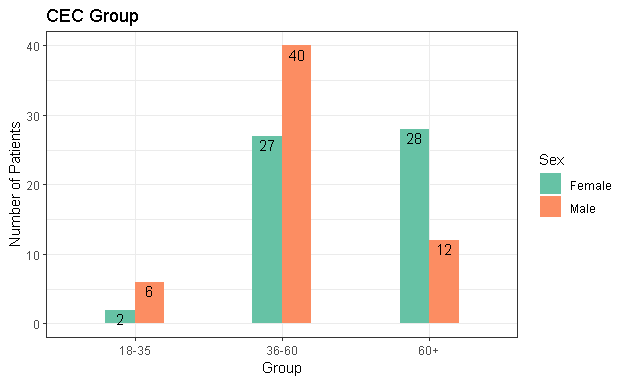


Figure S4 age-sex distribution in CEC group
